# Activation and execution of the hepatic integrated stress response by dietary essential amino acid deprivation is amino acid specific

**DOI:** 10.1096/fj.202200204RR

**Published:** 2022-06-12

**Authors:** William O. Jonsson, Emily T. Mirek, Ronald C. Wek, Tracy G. Anthony

**Affiliations:** ^1^ Department of Nutritional Sciences School of Environmental and Biological Sciences New Jersey Institute for Food Nutrition, and Health Rutgers University New Brunswick New Jersey USA; ^2^ Department of Biochemistry and Molecular Biology Indiana University School of Medicine Indianapolis Indiana USA

**Keywords:** dietary restriction, feeding, mammalian, polysomes, postprandial period, protein synthesis

## Abstract

Dietary removal of an essential amino acid (EAA) triggers the integrated stress response (ISR) in liver. Herein, we explored the mechanisms that activate the ISR and execute changes in transcription and translation according to the missing EAA. Wild‐type mice and mice lacking general control nonderepressible 2 (*Gcn2*) were fed an amino acid complete diet or a diet devoid of either leucine or sulfur amino acids (methionine and cysteine). Serum and liver leucine concentrations were significantly reduced within the first 6 h of feeding a diet lacking leucine, corresponding with modest, GCN2‐dependent increases in *Atf4* mRNA translation and induction of selected ISR target genes (*Fgf21*, *Slc7a5*, *Slc7a11)*. In contrast, dietary removal of the sulfur amino acids lowered serum methionine, but not intracellular methionine, and yet hepatic mRNA abundance of *Atf4*, *Fgf21*, *Slc7a5*, *Slc7a11* substantially increased regardless of GCN2 status. Liver tRNA charging levels did not correlate with intracellular EAA concentrations or GCN2 status and remained similar to mice fed a complete diet. Furthermore, loss of *Gcn2* increased the occurrence of ribosome collisions in liver and derepressed mechanistic target of rapamycin complex 1 signal transduction, but these changes did not influence execution of the ISR. We conclude that ISR activation is directed by intracellular EAA concentrations, but ISR execution is not. Furthermore, a diet devoid of sulfur amino acids does not require GCN2 for the ISR to execute changes to the transcriptome.

**Abbreviations**
AA: amino acid
*Actb*
: beta actin
AMPK: adenosine monophosphate kinase
ANOVA: analysis of variance
ATF4/*Atf4*
: activating transcription factor 4
BCAA: branched chain amino acid
Ctrl: control
DOC: deoxycholine
DTT: dithiothreitol
EAA: essential amino acid
ECL: enhanced chemiluminescence
EDTA: ethylenediaminetetraacetic acid
eIF2: eukaryotic initiation factor 2
ELISA: enzyme‐linked immunosorbent assay
FGF21/*Fgf21*
: fibroblast growth factor 21
GCN2/*Gcn2*
: general control nonderepressible 2
HEPES: 4‐(2‐hydroxyethyl)‐1‐piperazineethanesulfonic acid
HPLC: high performance liquid chromatography
ISR: integrated stress response
KO: knock out
LAT1: large neutral amino acid transporter
LD: leucine devoid
mRNA: messenger ribonucleic acid
mTORC1: mechanistic target of rapamycincomplex 1
PVDF: polyvinylidene difluoride
qPCR: quantitative polymerase chain reaction
RIPA: radio immune precipitation assay
RNase: ribonuclease
rpS6: ribosomal protein S6
SAA: sulfur amino acids
SAAD: sulfur amino acid devoid
SDS: sodium dodecyl sulfate
SEM: standard error of the mean
*Slc7a5*
: solute carrier 7a5, encodes
*Slc7a11*
: solute carrier7a11
tRNA: transfer ribonucleic acid
ULK1: Unc‐51 like autophagy activating kinase
WT: wild type
xCT: cystine‐glutamate antiporter
ZT: zeitgeber time

## 1 INTRODUCTION

Dietary restriction of either total protein or of specific essential amino acids (EAA) improves health span in rodents and extends lifespan in several experimental animal models.^1^, ^2^, ^3^, ^4^, ^5^ Mechanistically, removing or reducing one or more EAAs from the diet may create an intracellular amino acid imbalance in addition to reduced supply. At least two major sensing and signaling networks are impacted by this kind of EAA insufficiency: the integrated stress response (ISR) and the mechanistic target of rapamycin complex 1 (mTORC1). Both of these signal transduction networks are capable of sensing the missing or limiting AA but utilize different modes of detection.^6^, ^7^ The ISR regulator called general control nonderepressible 2 (GCN2) is activated by uncharged tRNAs^8^ or by ribosome stalling and collision.^9^ Activated GCN2 kinase then phosphorylates the eukaryotic initiation factor 2 (eIF2) on its α‐subunit, suppressing global protein synthesis and altering gene‐specific translation to maintain proteostasis control.^10^ While GCN2 is a sensor of EAA insufficiency, mTORC1 senses the availability of certain AAs in the cytosol and lysosome, triggering growth upon abundance or permitting reclamation during scarcity.^11^, ^12^, ^13^, ^14^, ^15^ While evidence for differential mTORC1 regulation in an amino acid specific manner exists,^16^, ^17^ evidence for amino acid‐specific control of the ISR and subsequent execution of gene targets is lacking.

Our previous work feeding mice diets restricted in the sulfur amino acids (SAA) showed that neither GCN2 nor the ISR effector, activating transcription factor 4 (ATF4), are required for sustained production of the stress‐responsive hepatokine, FGF21.^18^, ^19^ Other labs report that dietary restriction of leucine does not increase circulating FGF21 as strongly as dietary sulfur amino acid restriction.^20^ To test the hypothesis that control mechanisms within the ISR are differentially responsive to different forms or magnitudes of EAA insufficiency in a single meal, we fed mice diets that were devoid of either leucine or the sulfur amino acids (i.e., methionine and cysteine), and directly compared acute changes in the ISR regulator, core, effector and execution phases,^21^ alongside changes in tRNA charging, mTORC1 activity, and translational control in liver.

Herein, we show that execution of the ISR by leucine deprivation requires GCN2 whereas sulfur amino acid deprivation uses auxiliary or alternate means to effect and execute the ISR. We also observe that hepatic tRNA charging ratios do not explain GCN2 activation or ISR execution. While loss of *Gcn2* leads to greater mTORC1 signaling and ribosome collisions regardless of dietary EAA composition, these effects do not influence mRNA abundance or translational activity of selected ATF4 target genes. This information contributes novel information about how amino acid specific control of the ISR is executed in the liver of animals on a meal by meal basis.

## 2 MATERIALS AND METHODS

### 2.1 Animal experiments

All animal experiments were conducted in accordance with the Rutgers University Institutional Animal Care and Use Committee and NIH guidelines.^22^ To the best of our abilities, the reporting herein follows the ARRIVE guidelines.^23^ Young adult (approximately 3–4 months old) male C57BL/6J wild‐type (WT) mice and C57BL/6J mice lacking *Gcn2* (*Gcn2*KO)^24^ were habituated to a 12:12 h light:dark cycle with lights off at 1PM. Three to four mice per group were assigned to each group, a number that meets minimal requirements for conducting statistical comparisons. All mice, housed one animal per cage, had food removed during the last seven hours of the light photoperiod and then, upon light‐to‐dark transition, were freely provided one of three liquid experimental diets (described below) for the next six hours (i.e., first half of the dark photoperiod). During these experiments, pre‐fast, fasted and re‐fed body weights were measured. Food consumption was estimated by weighing the liquid diet containers prior to and immediately after each defined feeding time. Mice were killed by decapitation and blood and tissues were rapidly collected and snap frozen in liquid nitrogen. Trunk blood was centrifuged, and the serum was collected. All samples were stored at −80°C until further use.

### 2.2 Experimental diets

Three different liquid experimental diets (Dyets, Inc., Bethlehem, PA, USA) were used in this study: an AA complete control (Ctrl), a leucine devoid (LD), and a sulfur amino acid devoid (SAAD) diet, all of which were isocaloric and isonitrogenous (Table S1). All three diets were prepared by dissolving powered diets with water in equal parts by weight, per the manufacturer directions. Once fully in solution, diets were allowed to reach room temperature prior to being provided.

### 2.3 Liver and serum amino acid concentrations

Serum samples for AA concentration determination were prepared and analyzed by HPLC as previously described.^19^ Liver samples for hepatic AA concentration determination were prepared from approximately 50 mg frozen, crushed, liver tissue, and analyzed by HPLC as previously described.^25^ Subsequent peak identification and area under the curve calculations were performed using Agilent OpenLab (Agilent Technologies, Santa Clara, CA, USA).

### 2.4 Serum FGF21 concentrations

Serum concentrations of FGF21 were determined using a commercially available colorimetric sandwich ELISA (RD291108200R, BioVendor, Brno, Czech Republic) according to the manufacturer's instructions. Serum samples were diluted 10–20 times prior to analysis. The resulting absorbance was measured on a microplate reader (BioTek Synergy H1, Agilent Technologies) and FGF21 concentrations were calculated using the four‐parameter approach in R^26^ using RStudio^27^ and the drc package.^28^ For samples with concentrations below the limit of detection, we computed their concentration as equal to the lowest detected.

### 2.5 Polysome profiles

Polysome profile analysis was performed similar to what has been previously described,^29^, ^30^ with minor modifications as detailed below. Liver samples were prepared by homogenizing approximately 30 mg of frozen, crushed, liver tissue on ice in a 1:20 (w:v) ratio with ice‐cold lysis buffer (25 mM HEPES (pH 7.5), 100 mM KCl, 5 mM MgCl_2_, 1 mM DTT, 1% (v/v) Triton X‐100, 0.3% deoxycholate, 1x protease inhibitor (P8340, Sigma‐Aldrich, St. Louis, MO, USA), and 1200 U RNase Inhibitor (AM2696, Thermo Fisher Scientific, Waltham, MA, USA)) with disposable polypropylene pestles (12‐141‐364, Thermo Fisher Scientific). Samples were incubated on ice for 10 min prior to centrifugation at 10 000 *g* for 10 min at 4°C. Cleared supernatants were collected on ice and equal volumes of sample were loaded onto pre‐chilled 10%–50% (w/v) linear sucrose gradients (containing 25 mM Tris‐HCl (pH 8.0), 10 mM MgCl_2_ and 100 mM KCl prepared on a Gradient Station ip (BioComp Instruments, Inc., Fredericton, NB, Canada). Sucrose gradients with samples layered on top were weight‐equilibrated using 10% (w/v) sucrose solution and then subjected to centrifugation at 100 000 *g* for 3 h at 4°C in a swing bucket rotor (JS‐24.38, Beckman Coulter, Brea, CA, USA) centrifuge (Avanti J‐301, Beckman Coulter). Samples were then processed using a Gradient Station, with absorbance at 254 nm measured continuously (EM‐1 Econo UV Monitor, Bio‐Rad, Hercules, CA, USA). In a parallel set of liver lysates, 5 mM CaCl_2_ and 20 U DNase I (AM2222, Thermo Fisher Scientific) were added to the initial lysis buffer. Then, equal amounts of RNA (determined spectrophotometrically at 260 nm with a 1‐cm pathlength on a SpectraMax M2, Molecular Devices, San Jose, CA, USA) from mouse liver were incubated with 150 U Nuclease S7 micrococcal nuclease (10107921001, Sigma‐Aldrich) at 22°C using a temperature block (15‐600‐330, Thermo Fisher Scientific) for 45 min, after which the samples were treated with EDTA (5 mM final concentration) to stop digestion. Samples were then processed as described above. Undigested polysome profile data were analyzed and expressed as the ratio of area under the curve for the monosome peak and polysome peaks for each biological sample.

### 2.6 Targeted qPCR analysis of mRNA collected from polysome profiles

Targeted analysis of mRNA abundance in sucrose fractions was performed similar to what has previously been described,^30^ with minor modifications as indicated below. In brief, approximately 30 mg of frozen, crushed, liver tissue was homogenized and processed as described under polysome profiles. Upon processing samples through the Gradient Station, samples were fractionated (FC203B, Gilson, Middleton, WI, USA) and fractions (approximately 1 ml/fraction) corresponding to the 40S ribosomal subunits, 60S ribosomal subunits, and 80S monosomes were collected and combined into a “light” pool; fractions corresponding to trisomes, quadsomes, and penta/hexasomes were also collected and combined into a “heavy” pool (Figure S4B). Five nanograms of luciferase control RNA (L4561, Promega, Madison, WI, USA) was spiked‐in into each combined sample after which 3 ml of TRI Reagent RT‐Liquid Sample (RL311, Molecular Research Center, Inc., Cincinnati, OH, USA) was added for a final 1:1 ratio (sample:TRI Reagent) prior to mixing and snap freezing samples in liquid nitrogen. Light and heavy fractions for each sample were stored at −80°C. The following day, samples were thawed on ice and RNA was isolated using Direct‐zol RNA Miniprep kit (R2052, Zymo Research, Irvine, CA, USA) and vacuum filtered (EZ‐Vac Vacuum Manifold, S7000, Zymo Research) according to the manufacturer's recommendations. Sufficient quality and quantity of the isolated RNA was confirmed spectrophotometrically (NanoDrop One, Thermo Fisher Scientific) as well as by ethidium bromide gel electrophoresis. The cDNA was prepared from 1 µg of total RNA using reverse transcriptase (4368814, Thermo Fisher Scientific) according to the manufacturer's instructions. The resulting cDNA was used for SYBR green‐based qPCR analysis, with primers specified in Table S2, on a StepOnePlus system (Applied Biosystems). Relative transcript abundances in light and heavy pools for each sample were normalized to spike‐in luciferase RNA, calculated as 2^‐ΔCt, and then expressed as fold change of transcript in the heavy pool relative to the transcript abundance in respective light pool for each sample.

### 2.7 Total liver mRNA abundance by targeted qPCR

Approximately 15 mg of frozen, crushed, liver tissue was homogenized in 1 ml TRI Reagent RT (TR 118, Molecular Research Center, Inc.) using polypropylene pestles after which RNA was isolated using Direct‐zol RNA Miniprep kit according to the manufacturer's recommendations. Quality control, reverse transcription, and qPCR was performed as described above. Primer sequences are specified in Table S2. Gene expression analysis was performed using the 2^‐ΔΔCt‐method^31^ with *Actb* as reference and expressed as fold change relative to WT.Ctrl.

### 2.8 tRNA charging

Analysis of hepatic relative levels of charged and total tRNA isodecoders was performed using oligos specified in Table S2 and expressed as a fraction of the total tRNA isodecoder pool, as previously described,^25^, ^32^, ^33^ starting with 15 mg of frozen, crushed, liver tissue. To confirm this methodological approach, we ran a pilot experiment using samples from a previously published report^25^ which utilized halofuginone to pharmacologically induce the ISR via reduced prolyl‐tRNA charging (Figure S2).

### 2.9 Immunoblot analyses

Similar to previously described,^18^, ^19^ total protein was isolated from approximately 20 mg of frozen, crushed, liver tissue using a RIPA lysis solution consisting of 25 mM HEPES, 2 mM EDTA, 10 mM DTT, 50 mM sodium fluoride, 50 mM β‐glycerophosphate pentahydrate, 3 mM benzamidine, 1 mM sodium orthovanadate, 0.5% (w/v) sodium DOC, 1% (w/v) SDS, 1× protease inhibitor cocktail (P8340, Millipore‐Sigma), and 5 nM microcystin (33893, Millipore‐Sigma). Tissue samples were homogenized in a 1:30 (w:v) ratio with the above lysis solution on ice, after which samples were subjected to centrifugation for clarification at 10 000 *g* for 10 min at 4°C. Equal amounts of total protein were mixed 1:1 (v:v) with a 2× sample buffer solution (20% (v/v) glycerol, 60 mM Tris (pH 6.8), 2% (w/v) SDS, 0.01% (w/v) bromophenol blue, and 5% (v/v) β‐mercaptoethanol), after which samples were heated at 95°C for 4 min and subsequently stored at −20°C until further use. Gel electrophoresis was performed by separating equal amounts of protein (as determined using Pierce BCA Protein Assay, 23227, Thermo Fisher Scientific) by electrophoresis in SDS‐polyacrylamide gels and separated proteins were then transferred onto PVDF membranes. Protein‐bound membranes were blocked for one hour at room temperature before incubation with primary antibodies (Table S3) overnight. Immunoreactive bands were visualized by first incubating the membranes for one hour at room temperature with appropriate secondary antibodies (Table S3), followed by briefly incubating the membranes with ECL solution (RPN2235, Cytvia Amersham ECL Select Western Blotting Detection Agent, Cytvia, Marlborough, MA, USA) to image the targeted proteins (FluorChem M, ProteinSimple, San Jose, CA, USA). Densitometry was performed using AlphaView (v 3.4.0.0, ProteinSimple) and ImageJ (Fiji, v. 1.0).^34^ Values were normalized to either total protein (Coomassie) or, in the case of phosphorylated forms, to total and expressed as fold change compared to the WT.Ctrl group.

### 2.10 Statistical analyses

Analyses and visualization were performed using Microsoft Excel (v. 16.16.27), R (v. 4.0.3)^26^ and RStudio (v. 1.3.1093).^27^ Visualization and analyses in R were performed using the following packages: tidyverse,^35^ ggpubr,^36^ DescTools,^37^ car,^38^ and rstatix.^39^ Assumptions of normality and homoscedasticity were tested by Shapiro–Wilk and Levene's tests, respectively, and if necessary, data was log transformed to approach assumptions. Student's *t*‐test was used to compare the AA control group to each of the AA devoid diets. Two factor Analysis of variance (ANOVA) was used to assess main effects of diet and genotype, as further detailed in figure and table captions. Repeated‐measures ANOVA was used in time course comparisons. Unless otherwise specified, the significance level (*α*) was 0.05 and data are displayed as mean ± standard error of the mean (SEM).

## 3 RESULTS

### 3.1 Dietary EAA deprivation reduces intracellular concentrations of the limiting AA but does not alter tRNA charging

**FIGURE 1:**
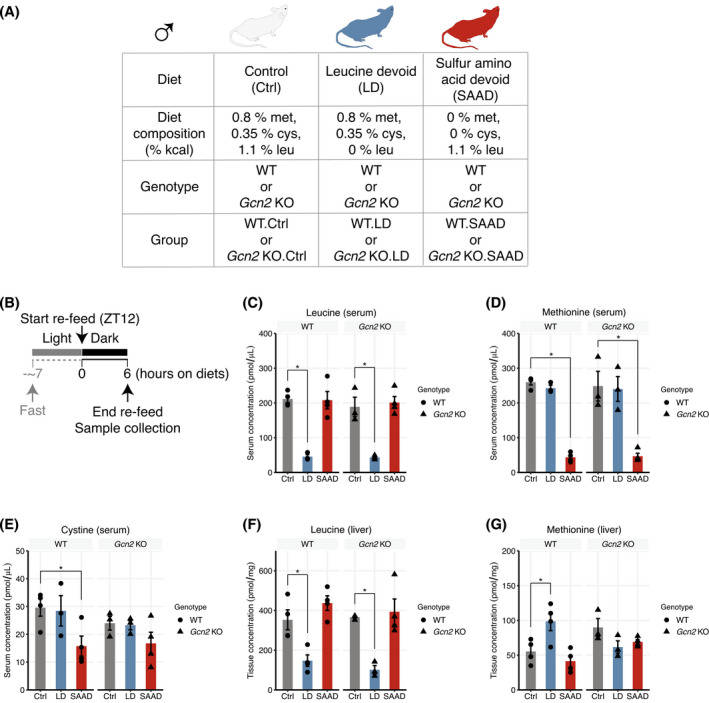
Male mice fed a leucine or sulfur amino acid devoid diet for six hours have reduced serum and liver amino acid levels. (A) Summary of diets and their detailed compositions (first two rows of table), genotypes (wild‐type or WT, and general control nonderepressible 2 knockout or *Gcn2*KO) (third row) and groups (bottom row). (B) Experimental outline with time above the outline indicating zeitgeber time (ZT) as well as light and dark photoperiods in relation to the start of the re‐feed. (C–G) Average serum and liver concentrations of (C, F) leucine and (D, G) methionine and (E) serum concentrations of cystine in WT and *Gcn2*KO mice refed a control (Ctrl), leucine devoid (LD) or sulfur amino acid devoid (SAAD) diet for six hours. *n* = 3–4/group. Displayed asterisks (*) indicates *p* < .05, as determined by Student's t‐test. Main effects of diet or genotype are detailed in Tables S4 and S5. Bar charts are presented as mean ± SEM, with individual values presented as dots.

Mice refed for six hours (Figure 1A,B) consumed food and regained weight regardless of genetic strain or diet consumed (Figure S1A,B). Circulating concentrations of EAA were altered in accordance with dietary intake (Table S4), with lower leucine in LD serum samples (Figure 1C) and lower methionine and cystine in SAAD serum samples (Figure 1D,E). Liver intracellular branched‐chain amino acid (BCAA) levels were altered in LD‐refed mice, independent of genotype (Figure 1F, Table S5). In contrast, liver methionine concentrations were unaltered in SAAD‐refed mice compared with mice fed Ctrl (Figure 1G). All amino acids with concentrations measured in serum and liver are reported in Tables S4 (serum) and S5 (liver intracellular).

**FIGURE 2:**
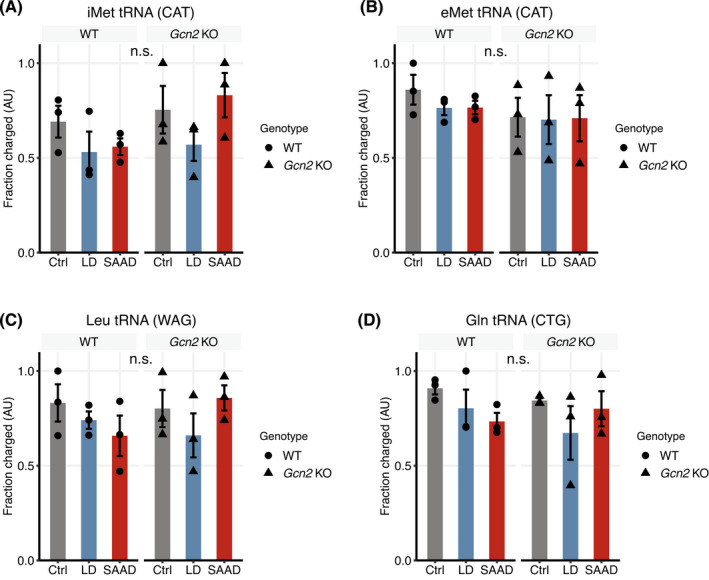
Liver tRNA charging levels in male mice fed a leucine or sulfur amino acid devoid diet for six hours. Average charged fractions of (A) initiator methionyl‐tRNA (iMet), (B) elongator methionyl‐tRNA (eMet), (C) leucyl‐tRNA and (D) glutamyl‐tRNA in livers of wild‐type (WT) and general control nonderepressible 2 knockout (*Gcn2*KO) mice refed a control (Ctrl), leucine devoid (LD) or sulfur amino acid devoid (SAAD) diet for six hours. *n* = 3–4/group. n.s. indicates no statistical difference at *α* = 0.05, as determined by Student's *t*‐test. Bar charts are presented as mean ± SEM, with individual values presented as dots.

Based on differences in the magnitude of reduction in concentrations of liver leucine versus liver methionine, we examined how tRNA charging, the canonical GCN2 trigger under EAA deprivation, was impacted. We first validated our approach and the method using liver samples from halofuginone‐treated mice^25^ to confirm the expected reduced prolyl tRNA charging (Figure S2). Then, the relative charged fraction of isodecoders for leucine, methionine, and glutamine in livers were measured in the Ctrl, LD, and SAAD diet groups in both WT and *Gcn2*KO mice. In the case of leucine, we measured the Leu^WAG^ isodecoder, since it corresponds with one of the more frequently used isodecoders,^40^, ^41^ and in the case of methionine, we measured the relative charged fraction of both the initiator (i) and elongator (e) Met^CAT^ isodecoders. No significant declines in the relative charged fractions of these isodecoders were detected in the livers of WT or *Gcn2*KO mice fed LD or SAAD (Figure 2A–C). In light of recent data showing that tRNA^Gln^ disproportionately accumulates in its uncharged form during AA starvation,^32^ we also measured relative levels of charged Gln^CTG^, but again, did not detect any differences between groups (Figure 2D). Taken together, these data show that refeeding mice a diet devoid in either leucine or methionine plus cysteine did not significantly lower tRNA charging even when liver concentrations of the targeted AA were reduced.

### 3.2 Activation of the ISR is AA specific

**FIGURE 3:**
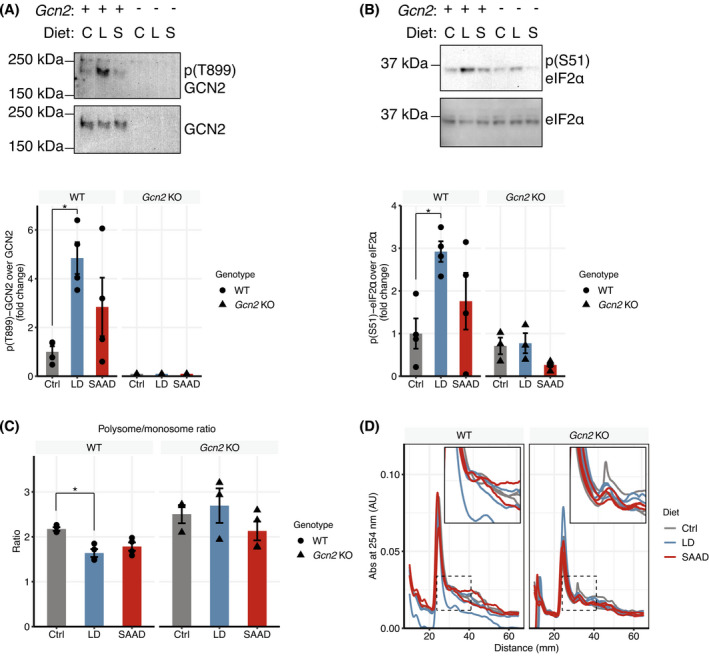
Hepatic ISR signaling during dietary EAA deprivation varies as a function of the missing amino acid and impacts translational control. Quantification and representative western blots, based on samples from wild‐type (WT) or *Gcn2* knockout (Gcn2KO) mice refed either a control (Ctrl or C), leucine devoid (LD or L) or sulfur amino acid devoid (SAAD or S) diet for six hours, of hepatic (A) phosphorylated (T899)‐GCN2 over GCN2 and (B) phosphorylated (S51)‐eIF2α over eIF2α. (C) Average polysome over monosome ratios in liver samples from mice refed for six hours. (D) Individual polysome profiles from ribonuclease‐treated liver samples from WT and *Gcn2*KO mice refed for six hours. Distance (in millimeters, mm) corresponds to distance from top of centrifugation tubes, as absorbance (Abs) was measured along the length of tubes. Solid black squares are enlarged from areas indicated by dashed black squares for each genotype. *n* = 3–4/group. Displayed asterisks (*) indicates *p* < .05, as determined by Student's *t*‐test. n.s. indicates no statistical difference at *α* = 0.05. Bar charts are presented as mean ± SEM, with individual values presented as dots.

We next surveyed hepatic ISR signaling in response to dietary AA deprivation. First, we confirmed the complete absence of GCN2 in the livers of *Gcn2*KO mice (Figure 3A). We then assessed induction of GCN2 phosphorylation (at T899), the ISR regulator activated by EAA deprivation, in both LD and SAAD liver samples. Only in LD‐fed WT mice was there increased GCN2 and eIF2α phosphorylation (at S51) (Figure 3A,B), with a clear correlation between GCN2 and eIF2α phosphorylation (Figure S3A). Concomitant with these changes, we also noted a reduced polysome to monosome ratio in WT mice fed LD (Figures 3C and S4B), indicating a global reduction in mRNA translation that was GCN2‐dependent. Dietary SAAD did not significantly alter any of these parameters in liver. We also noted an increased abundance of ribonuclease‐resistant disomes in livers from *Gcn2*KO mice (Figure 3D). In tissue samples treated with RNase and then centrifuged across a sucrose gradient, ribosomes bound to mRNA are detected at absorbance 254 nm as a single monosome peak. The presence of a second peak positioned to the right of the large monosome peak (see inset in upper right of Figure 3D) suggests the presence of a population of ribosomes that are so close as to leave no unprotected mRNA between them, resulting in a RNase‐resistant disome. This observation suggests consequences at the elongation step in the absence of *Gcn2* which may be indicative of increased ribosome collision independent of diet.^42^ We then turned to hepatic mTORC1 signaling and assessed its control in the presence versus absence of GCN2 by measuring the phosphorylation of mTOR (at S2448) and the phosphorylation of ribosomal protein S6 (rpS6, at S235/236) in liver samples. We noted a robust increase in both mTOR and rpS6 phosphorylation in *Gcn2*KO livers, independent of diet (Figure S3B,C). These effects were not associated with changes in the phosphorylation of AMPK (at T172) (Figure S3D), indicating no major changes in energy state among the diet groups or between the strains. Furthermore, mTORC1‐mediated inhibitory phosphorylation of ULK1 (at S757), a central protein in autophagy induction remained unaltered (Figure S3E).

### 3.3 LD and SAAR increase ATF4 synthesis and its gene targets differently

**FIGURE 4:**
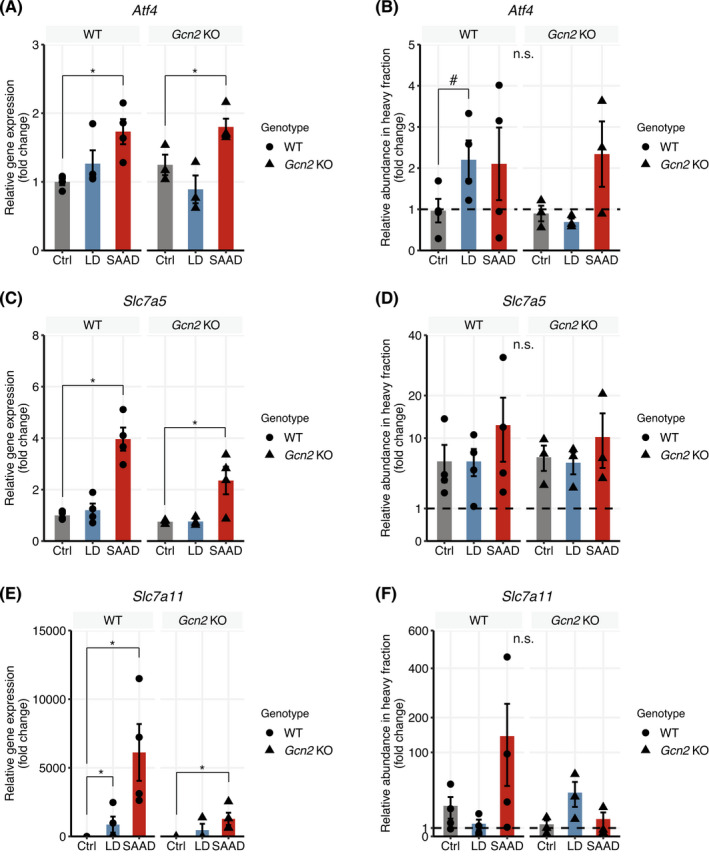
Male mice fed a leucine or sulfur amino acid devoid diet for six hours show altered transcription and translation of stress‐responsive transcripts in liver. Relative total transcript abundance (A, C, and E) and relative abundance in heavy polysome fractions (B, D, and F) of stress‐responsive transcripts in liver samples from wild‐type (WT) and *Gcn2* knockout (*Gcn2*KO) mice refed either a control (Ctrl), leucine devoid (LD) or sulfur amino acid devoid (SAAD) diet for six hours. Measured transcripts were (A, B) *Atf4*, (C, D) *Slc7a5* and (E, F) *Slc7a11*. Dashed lines in B, D, and F indicates the relative level (set to one) for the corresponding light fractions for each group to which the heavy fraction transcript abundance was compared to and expressed as fold change over. *n* = 3–4/group. Displayed asterisks (*) indicates *p* < .05 and hash (#) indicates *p* = .09, as determined by Student's *t*‐test. n.s. indicates no statistical difference at *α* = 0.05. Bar charts are presented as mean ± SEM, with individual values presented as dots.

Activation of the ISR elicits two major outcomes: first, a reduction in general protein synthesis (as seen in Figure 3C); and second, increased ATF4 synthesis, directing transcription of its target genes. Considering the observed EAA‐specific ISR signaling in liver, we measured mRNA abundance of *Atf4* in both whole liver lysates and across polysome profiles. We noted a significant increase in hepatic *Atf4* in both WT and *Gcn2*KO mice fed SAAD relative to their own Ctrl (Figure 4A). In contrast, hepatic mRNA abundance of *Atf4* in both WT and *Gcn2*KO mice fed LD remained similar to their own Ctrl. We then examined two ATF4 target genes, *Slc7a5* (encoding LAT1, involved in transporting leucine) and *Slc7a11* (encoding the cystine transporter, xCT). In both cases, the same pattern emerged, increased mRNA abundance in the livers of SAAD‐fed mice but not LD‐fed mice (Figure 4C,E). Next, we measured mRNA levels of *Atf4* and its target genes across polysomes profiles as a measure of translational activity. Sucrose fractions containing highly translated transcripts (“heavy” i.e., mRNAs associated with polysomes) were pooled and mRNA levels in the combined fractions were expressed relative to that measured in pooled sucrose factions containing lowly translated transcripts (“light” i.e., mRNAs associated with 40S, 60S, and 80S ribosomes) (Figure S4B). The transcript abundance of *Atf4* and its target genes in the heavy pool relative to the respective light pool for each individual sample was then calculated, allowing for diet comparisons in WT and *Gcn2*KO mice. Using this approach, we observed WT mice fed LD showed increased hepatic ATF4 synthesis, reflected as a doubling of *Atf4* abundance in polysomes relative to Ctrl (Figure 4B). Loss of *Gcn2* precluded increased translational activity of hepatic *Atf4* in LD‐fed mice. Interestingly, average *Atf4* translation also doubled in the livers of both WT and *Gcn2*KO mice fed SAAD, but reached statistical significance only in *Gcn2*KO mice. The translational activity of both *Slc7a5* and *Slc7a11* showed no significant changes and generally followed a pattern similar to their mRNA abundance, consistent with the idea that transcriptional induction by ATF4 is a key regulator of increased production of AA transporters (Figure 4D,F). Furthermore, *Gcn2* deletion did not affect translational activities of *Slc7a5* and *Slc7a11*. As a non‐ISR control gene, we measured mRNA abundance of the housekeeping gene, *Actb*, in liver lysates and in pooled sucrose fractions and observed that both total and polysomal mRNA remained similar across all treatment groups (Figure S4C,D).

### 3.4 Transcriptional induction of *Fgf21* in liver elevates serum FGF21 during dietary EAA deprivation

**FIGURE 5:**
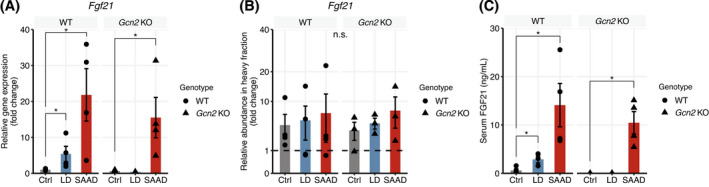
Male mice fed a leucine or sulfur amino acid devoid diet for six hours showed increased serum FGF21 as a function of increased *Fgf21* mRNA levels in liver. (A) Total relative *Fgf21* mRNA abundance in livers of wild‐type (WT) and *Gcn2* knockout (*Gcn2*KO) mice refed a control (Ctrl), leucine devoid (LD) or sulfur amino acid devoid (SAAD) diet for six hours. (B) Relative abundance of *Fgf21* in heavy fractions in liver samples from mice refed for six hours. (C) Serum concentrations of FGF21 in WT and *Gcn2*KO mice refed either a Ctrl, LD, or SAAD diet for six hours. Dashed line in B indicate the relative level (set to one) for the corresponding light fractions for each group to which the heavy fraction transcript abundance was compared to. *n* = 3–4/group. Displayed asterisks (*) indicates *p* < .05, as determined by Student's *t*‐test. n.s. indicates no statistical difference at *α* = 0.05. Bar charts are presented as mean ± SEM, with individual values presented as dots.

We also assessed liver production of FGF21, a hepatokine central in the systemic response to dietary and pharmaceutical forms of protein dilution and EAA deprivation.^19^, ^43^, ^44^, ^45^ We found that LD increased total *Fgf21* mRNA abundance in liver of WT but not *Gcn2*KO mice (Figure 5A). In contrast, SAAD‐fed mice increased *Fgf21* independent of *Gcn2*. Relative abundance of *Fgf21* in polysomes remained similar across treatment groups (Figure 5B). Serum concentrations of FGF21 strongly correlated with mRNA levels (Pearson's *R* = .92, *p* < .05) but showed no relationship with translational activity (Figure 5C). Circulating FGF21 was modestly increased by LD only in WT mice. In contrast, SAAD substantially increased serum FGF21 in both WT and *Gcn2*KO mice and about four‐fold greater than WT mice fed LD (Figure 5C).

## 4 DISCUSSION

The ability of an animal to maintain proteostasis control in the face of reduced nutrients is important for overall health and survival. Regulation of protein biogenesis is central for proteostasis control, and the supply and balance of AAs are its most basic cues. Among the control mechanisms activated by changes in the supply and balance of EAAs, the ISR has emerged as a key regulator. However, many questions exist about how the ISR is executed under different forms of EAA deprivation. The current study was designed to examine in a single meal setting to what extent does the hepatic ISR tailor its effects, either in translational activity or transcriptional execution, according to the dietary limiting EAA.

We examined the ISR after six hours of feeding in order to allow sufficient time for both transcriptional as well as translational changes to occur in response to reduced circulating and intracellular amino acid concentrations. Previous studies by our group show that one hour of AA deprivation by diet or drug alter signaling and mRNA translation but not mRNA expression levels^30^, ^46^, ^47^ while six hours captures changes in protein synthesis as well as induction of ISR gene targets in mouse liver.^25^, ^48^ Feeding diets devoid of leucine or methionine significantly lower intracellular levels of leucine but not methionine. The magnitude of these intracellular changes corresponds with activation of the ISR regulator, GCN2, and phosphorylation of eIF2. Furthermore, while leucine concentrations in serum and liver correlate with each other, methionine concentrations do not. Since methionine serves a critical role in many different biological processes, including translation initiation, greater capacity may exist to maintain intracellular levels of methionine than leucine.

It is interesting to note that despite achieving significant reductions in serum and/or liver amino acid levels, an accumulation of uncharged tRNAs specific to the depleted EAA did not occur. These findings contrast with those observed in drug‐induced AA stress in mice.^25^ Perhaps dietary EAA deprivation is less potent in reducing tRNA charging compared to a drug like halofuginone which effectively depletes hepatic prolyl‐tRNA charging.^25^ Or perhaps rather than reducing the charging of individual tRNAs, dietary EAA deprivation alters charging across many different tRNA species. This idea aligns with studies which suggest that tRNA pools act in a dynamic, concerted, fashion during AA starvation to maintain translational fidelity, a crucial factor in proteostasis control.^49^, ^50^ Alternatively, dietary EAA deprivation may induce changes in a more localized fashion, resulting in a diluted signal in these measurements. Further investigation of these differences and how they may relate to sparing of tRNA charging are warranted, necessitating a broader view of the isodecoder landscape.^32^, ^51^

This study was designed to determine relative differences in ISR activation, translational activity, and transcriptional execution upon refeeding different EAA devoid diets in both WT and *Gcn2*KO mice. While ISR activation correlated with intracellular concentrations of the dietary limiting EAA, ISR execution did not. Indeed, *Atf4* and its target genes were significantly increased in mRNA abundance by SAAD even though very minor to modest activation of the ISR occurred. It is difficult to know if changes in mRNA abundance or translation of *Slc7a5* (encoding LAT1, which imports large neutral amino acids including leucine and methionine) and *Slc7a11* (encoding xCT, which imports cystine) were consequential in buffering and/or sparing intracellular methionine requirements.^52^ In the case of leucine, because it shares the low capacity LAT1 transporter with methionine and other AAs, increased transport capacity may exacerbate instead of alleviate the dietary imbalance.^53^ In support of this, LD‐fed WT mice had increased liver methionine, among other EAAs (Table S5). Considering both the ISR and mTOR can influence the expression, cellular localization and activity of amino acid transporters such as xCT,^54^, ^55^, ^56^ future exploration in these areas is warranted. Furthermore, because prolonged fasting increases eIF2 phosphorylation and reduces mTORC1 signaling,^57^ it would be interesting to directly compare feeding an EAA devoid diet to the prolonged fasted state; this is something to pursue in future studies.

We also noted that mRNA abundance of *Fgf21* correlated with serum FGF21, but increased FGF21 by LD was dependent on GCN2 whereas SAAD was not. Possibly related to this, Welles and colleagues previously showed that conditions of elevated mTORC1 signaling during refeeding suppresses *Fgf21* translation without changing transcript levels.^58^ With the increased mTORC1 activation in *Gcn2*KO mice, a similar mechanism might be at play in our study but this remains to be further explored.

Overall, we show that both LD and SAAD engage signaling networks and translational control in the liver of mice, but to different degrees. This likely reflects AA‐specific control mechanisms that serve to spare or rescue the intracellular limiting AA to maintain homeostasis. Our work may provide retrospective mechanistic clarity of other previous studies on EAA restriction or deprivation in mice.^20^, ^59^ Specifically, we show that in liver, dietary leucine deprivation engages canonical ISR signaling to a greater extent than dietary sulfur amino acid deprivation. However, neither devoid diet markedly reduced tRNA charging of individual isodecoders. These observations point to the existence of additional means of ISR activation beyond accumulation of uncharged tRNAs. Loss of *Gcn2* results in unleashing of mTORC1,^25^, ^30^, ^60^ and increased abundance of ribonuclease resistant disomes, suggesting ribosomal collision. Considering that the execution of the ISR can occur in the absence of increased eIF2α phosphorylation,^18^ it stands to reason that induction of ATF4 target genes may occur through other auxiliary inputs including mTORC1 and N6‐methyladenosine marks when GCN2 is absent.^56^, ^61^, ^62^ Further examination of these and other control mechanisms activated by altered dietary EAA supply to different tissues and at different timepoints is warranted. Considering that both individual food products (e.g, gelatin) and dietary plans (e.g., protein dilution, strict veganism) likely engage the ISR, these results are applicable to the fields of proteostasis and dietary restriction.

## AUTHOR CONTRIBUTIONS

William O. Jonsson and Tracy G. Anthony conceived and designed the study. William O. Jonsson and Emily T. Mirek conducted experiments. William O. Jonsson analyzed and visualized data. William O. Jonsson, Ronald C. Wek, and Tracy G. Anthony interpreted data. William O. Jonsson and Tracy G. Anthony and wrote the original draft manuscript. William O. Jonsson, Emily T. Mirek, Ronald C. Wek, and Tracy G. Anthony critically reviewed and edited the manuscript and approved of submitted version.

## FUNDING INFORMATION

National Institute of Health (DK109714 to TGA and RCW), the United States Department of Agriculture (NIFA/Hatch NC1184 award to TGA), and the Rutgers Center for Lipid Research (to WOJ).

## DISCLOSURES

TGA and RCW have served as scientific consultants for HiberCell Inc., WOJ and ETM declare no conflicts of interest.

## Supporting information

Fig S1

Fig S2

Fig S3

Fig S4

Table S1

Table S2

Table S3

Table S4

Table S5

## Data Availability

The data that support the findings in this study are either available in the main text, Supporting Information, or from the corresponding author upon request.
