# Supplementary material for: Activation and execution of the hepatic integrated stress response by dietary essential amino acid deprivation is amino acid specific
Source: FASEB J. 2022 Jun 12;36(7):e22396. doi: 10.1096/fj.202200204RR (PMC9204950; doi:10.1096/fj.202200204RR)
Supplement: Supplementary file 1 — Fig S1 [file FSB2-36-0-s007.pdf]

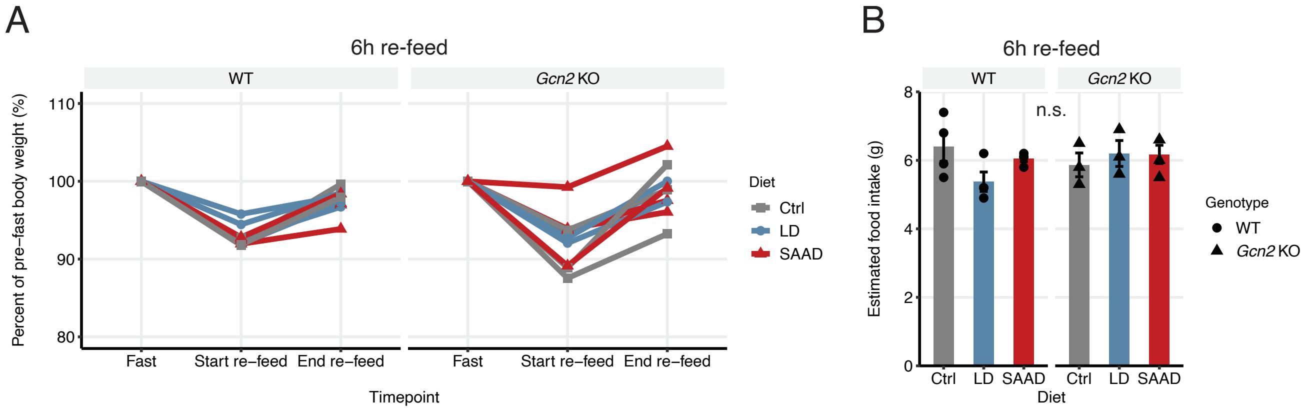

Figure S1. Male mice provided diets devoid of leucine or the sulfur amino acids following a seven hour period of food deprivation consume their food and regain their body weight within six hours of feeding.

Changes in body weight and food consumption in wild-type (WT) and general control nonderepressible 2 knockout (*Gcn2*KO) mice refed a control (Ctrl), leucine devoid (LD) or sulfur amino acid devoid (SAAD) diet for six hours.

(A) Body weight change for individual WT and *Gcn2*KO mice refed for six hours.

(B) Average estimated food intake in WT and *Gcn2*KO mice refed for six hours.

n = 3-4/group. n.s. indicates no statistical difference at $\alpha = 0.05$. Bar charts are presented as mean ± SEM, with individual values presented as dots. Dots connected by lines represent values from one individual animal.
