# Supplementary material for: Activation and execution of the hepatic integrated stress response by dietary essential amino acid deprivation is amino acid specific
Source: FASEB J. 2022 Jun 12;36(7):e22396. doi: 10.1096/fj.202200204RR (PMC9204950; doi:10.1096/fj.202200204RR)
Supplement: Supplementary file 2 — Fig S2 [file FSB2-36-0-s004.pdf]

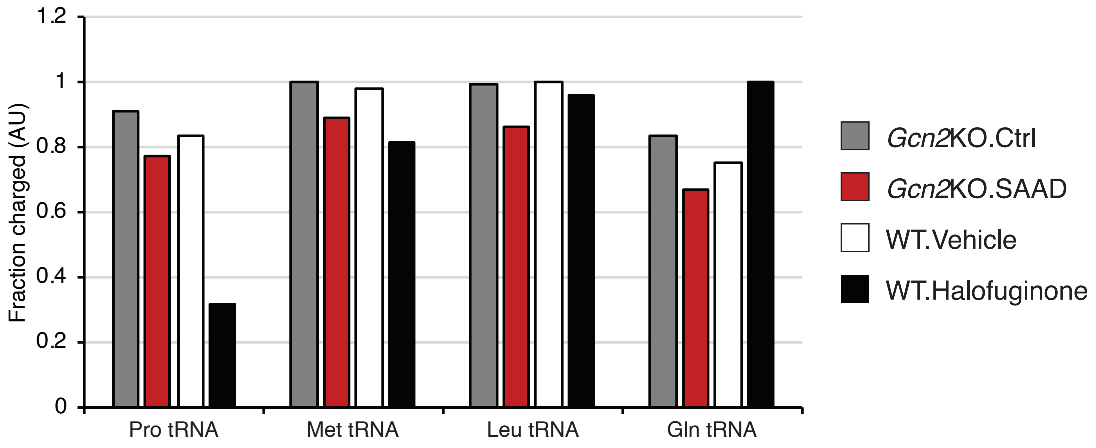

Figure S2. Confirmatory tRNA charging analysis using vehicle and halofuginone-treated liver samples. Charged fractions of prolyl-tRNA, initiator methionyl-tRNA, leucyl-tRNA, and glutamyl-tRNA in liver samples from *Gcn2* knockout (*Gcn2*KO) mice refed either a control (Ctrl) or sulfur amino acid devoid (SAAD) diet for six hours, or wild-type (WT) mice injected twice with 0.5 % (v/v) DMSO (vehicle) or 0.5 mg/kg halofuginone. n = 1/treatment.
