# Supplementary material for: Activation and execution of the hepatic integrated stress response by dietary essential amino acid deprivation is amino acid specific
Source: FASEB J. 2022 Jun 12;36(7):e22396. doi: 10.1096/fj.202200204RR (PMC9204950; doi:10.1096/fj.202200204RR)
Supplement: Supplementary file 3 — Fig S3 [file FSB2-36-0-s003.pdf]

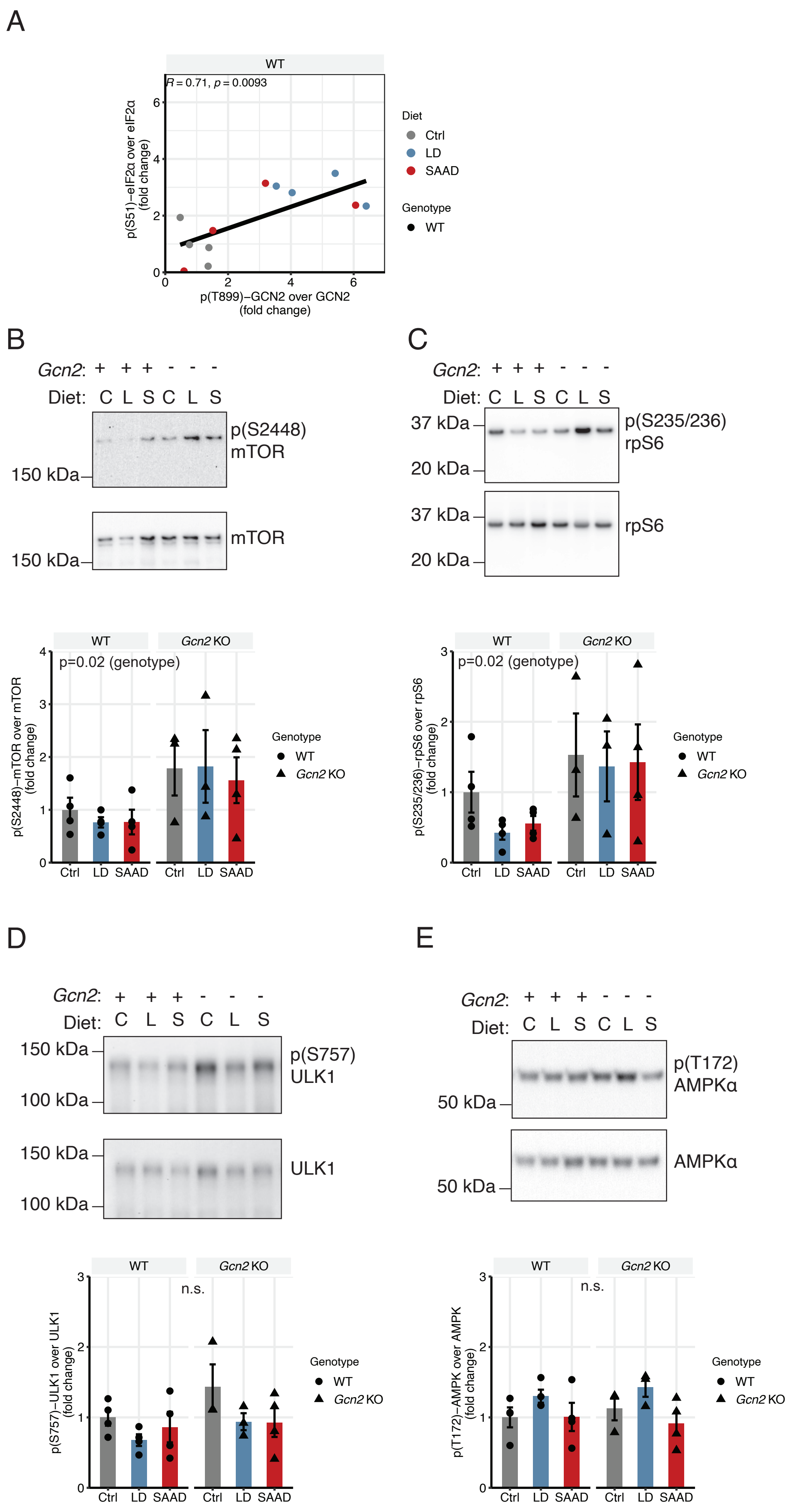

Figure S3. Male mice provided diets devoid of leucine or the sulfur amino acids for six hours altered hepatic ISR and mTORC1 signaling.

(A) Correlation between the phosphorylation of eIF2α phosphorylation and GCN2 phosphorylation in wild-type (WT) mice refed either a control (Ctrl), leucine devoid (LD) or sulfur amino acid devoid (SAAD) diet for six hours. Displayed Pearson correlation coefficient (R) are for all biological samples combined, with dots in scatter plots being values from individual animals.

(B-D) Quantification and representative western blots, based on samples from WT or *Gcn2* knockout (*Gcn2*KO) mice refed either a Ctrl (or C), LD (or L) or SAAD (or S) diet for six hours.

(B) Phosphorylated (S2448)-mTOR over mTOR in liver.

(C) Phosphorylated (S235/236)-rpS6 over rpS6 in liver.

(D) Phosphorylated (S757) ULK1 over ULK1 in liver.

(E) Phosphorylated (T172) AMPKα over AMPKα in liver.

n = 3-4/group. Main effect of genotype was detected by two way ANOVA. n.s., indicates no effects of genotype, diet or treatment at α = 0.05. Bar charts are presented as mean ± SEM, with individual values presented as dots.
