# Supplementary material for: Activation and execution of the hepatic integrated stress response by dietary essential amino acid deprivation is amino acid specific
Source: FASEB J. 2022 Jun 12;36(7):e22396. doi: 10.1096/fj.202200204RR (PMC9204950; doi:10.1096/fj.202200204RR)
Supplement: Supplementary file 4 — Fig S4 [file FSB2-36-0-s009.pdf]

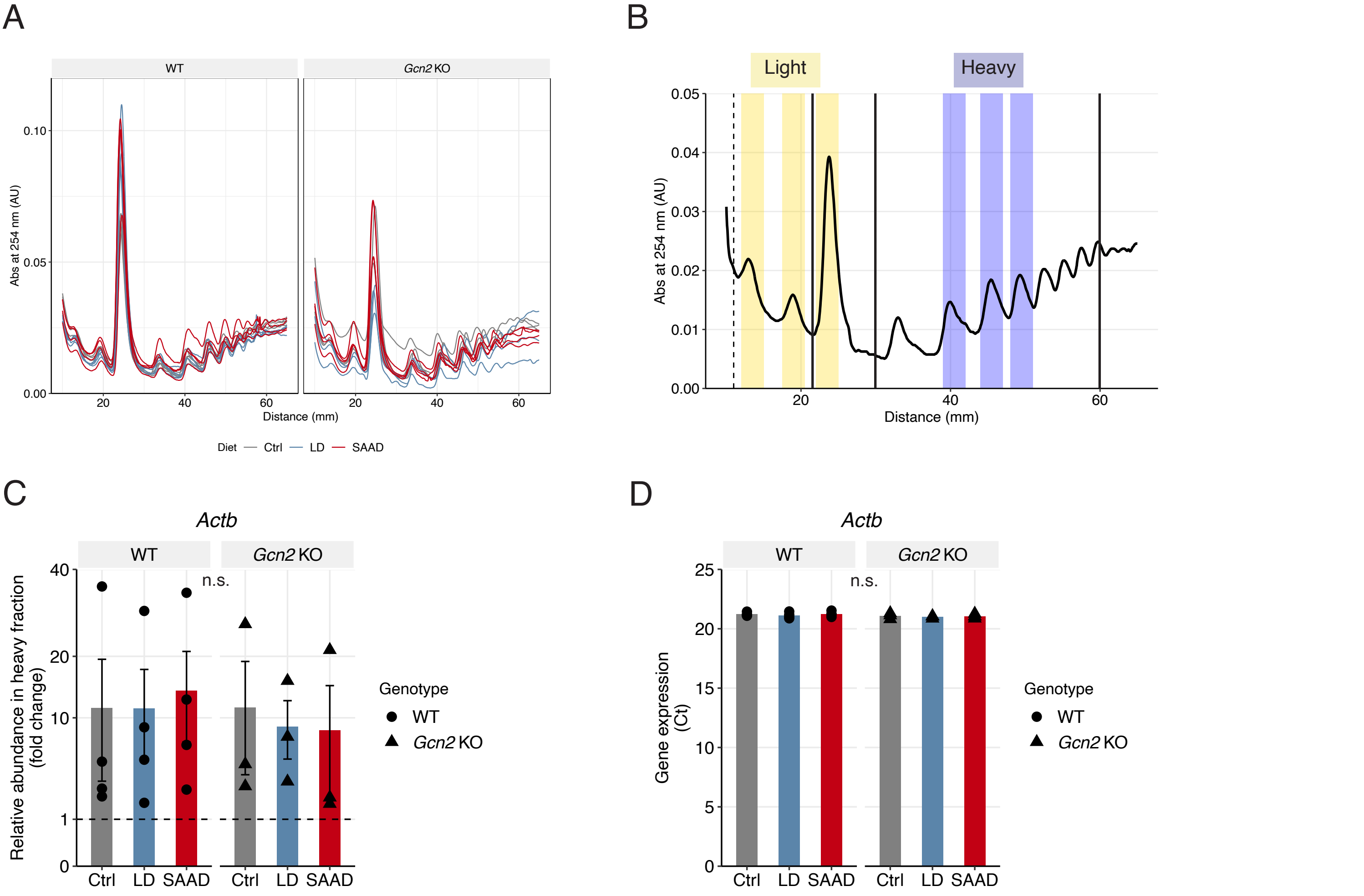

Figure S4. Male mice provided diets devoid of leucine or the sulfur amino acids for six hours showed global and transcript-specific changes in translation.

(A) Individual polysome profiles from liver samples from wild-type (WT) and *Gcn2* knockout (*Gcn2*KO) mice refed either a control (Ctrl), leucine devoid (LD) or sulfur amino acid devoid (SAAD) diet for six hours. Distance (in millimeters, mm) corresponds to distance from top of centrifugation tubes, as absorbance (Abs) was measured along the length of tubes.

(B) Example polysome profile with indicated “light” (yellow) and “heavy” (blue) fractions that were pooled for the targeted qPCR analysis. The area between the two left-most solid lines illustrates the area designated as the monosome area under the curve for the polysome to monosome ratio analysis; the area between the two right-most solid lines indicate the area designated as the polysome area for the same analysis.

(C) Relative transcript abundance of *Actb* in heavy sucrose fractions in WT and *Gcn2*KO mice refed either a Ctrl, LD or SAAD diet for six hours. Dashed line in B indicate the relative level (set to one) for the corresponding light fractions for each group to which the heavy fraction transcript abundance was compared to.

(D) Average cycle threshold (Ct) of *Actb* in WT and *Gcn2*KO mice refed either a Ctrl, LD or SAAD diet for six hours. n = 3-4/group. "n.s." indicates no statistical difference at $\alpha = 0.05$, as determined by Student’s t-test. Bar charts are presented as mean ± SEM, with individual values presented as dots.
