# Supplementary material for: Activation and execution of the hepatic integrated stress response by dietary essential amino acid deprivation is amino acid specific
Source: FASEB J. 2022 Jun 12;36(7):e22396. doi: 10.1096/fj.202200204RR (PMC9204950; doi:10.1096/fj.202200204RR)
Supplement: Supplementary file 5 — Table S1 [file FSB2-36-0-s005.pdf]

Table S1. Composition of experimental diets.

Diet	Control diet		Leucine devoid		Sulfur amino acid devoid	
Produer	Dyets Inc.		Dyets Inc.		Dyets Inc.	
Product#	710179		710182		710184	
Unit	% kcal		% kcal		% kcal	
Carbohydrate	64.4		64.6		64.6	
Fat	17.8		17.8		17.8	
Protein	17.8		17.6		17.6	
Unit	g/L	kcal/g	g/L	kcal/g	g/L	kcal/g
L-Alanine	0.87		1.34		1.34	
L-Arginine (free base)	3		3		3	
L-Asparagine H2O	1.5		1.5		1.5	
L-Aspartic Acid	0.87		1.56		1.56	
L-Cystine	0.87		0.87		0	
L-Glutamic acid	9.9		10.68		10.68	
Glycine	5.77		6.17		6.17	
L-Histidine (free base)	1.1		1.1		1.1	
L-Isoleucine	2		2		2	
L-Leucine	2.75		0		2.75	
L-Lysine HCl	4.5		4.5		4.5	
L-Methionine	2		2		0	
L-Phenylalanine	1.9		1.9		1.9	
L-Proline	0.87		0.87		0.87	
L-Serine	0.87		0.87		0.87	
L-Threonine	2		2		2	
L-Tryptophan	0.45		0.45		0.45	
L-Tyrosine	1.24		1.24		1.24	
L-Valine	2		2		2	
Total L-AA	44.46	177.84	44.05	176.2	43.93	175.72
Sucrose	156.86	627.4	157.34	629.4	157.39	629.6
Maltose Dextrin	0	0	0	0	0	0
Cellulose	13	0	13	0	13	0
Soybean Oil	19.8	178.2	19.8	178.2	19.8	178.2
TBHQ	0.004	0	0.004	0	0.004	0
Salt Mix #210033	9.21	6.35	9.21	6.35	9.21	6.35
Sodium Bicarbonate	1.95	0	1.95	0	1.95	0
Vitamin Mix #310025	2.6	10.2	2.6	10.2	2.6	10.2
Xanthan Gum	3	0	3	0	3	0
Choline Bitartrate	0.66	0	0.66	0	0.66	0
Total	251.574	1000.01	251.64	1000.29	251.574	1000.01

Each diet was prepared by mixing to a final concentration of 251.6 g/L in cold water
