# Supplementary material for: Activation and execution of the hepatic integrated stress response by dietary essential amino acid deprivation is amino acid specific
Source: FASEB J. 2022 Jun 12;36(7):e22396. doi: 10.1096/fj.202200204RR (PMC9204950; doi:10.1096/fj.202200204RR)
Supplement: Supplementary file 6 — Table S2 [file FSB2-36-0-s006.pdf]

Table S2. List of oligos

Primers (5' to 3')		
Target	Forward primer	Reverse primer
Actb	GGCTGTATTCCCCTCCATCG	CCAGTTGGTAACAATGCCATGT
Atf4	GAGTTTGACTTCGCTCTGTTTCGAATGGATGA	CAATTGGGTTCACTGTCTGAGGGGGCTCCTTATTAG
Fgf21	AGCATACCCCATCCCTGACT	AGGAGACTTTCTGGACTGCG
Slc7a11	ATCTCCCCCAAGGGCATACT	GAGCAGTTCCACCCAGACTC
Slc7a5	CTGGTCTTCGCCACCTACTT	GCCTTTACGCTGTAGCAGTTC
luciferase	CCAGGGATTTCAGTCGATGT	AATCTCACGCAGGCAGTTCT
mouse-Leu-tRNA	GGTAGYGTGGCCGAGCG	GAGAATTCCATGGYGGTGGG
mouse-iMet-tRNA	AGCAGAGTGGCGCAGCG	GAGAATTCCATGGTAGCAGAGGATGGTTTCG
mouse-eMet-tRNA	GCCTCSTTAGCGCAGTAGGTAG	GAGAATTCCATGGTGCCCCSTS
mouse-Gln-tRNA	GGTTCCATGGTGTAATGGTNAGCACTCTG	GAGAATTCCATGGAGGTTCCACCGAGATTTG
mouse-Pro-tRNA	GGCTCGTTGGTCTAGGGGTA	GAGAATTCCATGGGGGCTCGTCC
yeast-Phe-tRNA	GCGGAYTTAGCTCAGTTGGGAGAG	GAGAATTCCATGGTGCGAAYTCTGTGG
Other oligos (5' to 3')		
5-adenylated adaptor	5'-/5rApp/TGGAATTCTCGGGTGCCAAGG/3ddC/-3'	
RT oligo	5'-GCCTTGGCACCCGAGAATTCCA-3'	
