# Supplementary material for: Activation and execution of the hepatic integrated stress response by dietary essential amino acid deprivation is amino acid specific
Source: FASEB J. 2022 Jun 12;36(7):e22396. doi: 10.1096/fj.202200204RR (PMC9204950; doi:10.1096/fj.202200204RR)
Supplement: Supplementary file 7 — Table S3 [file FSB2-36-0-s001.pdf]

Table S3. List of antibodies

Antibody	Supplier	Product number	Antibody dilution
Anti-AMPKα	Cell Signaling Technologies	2532	1:1000
Anti-p(T172)-AMPKα	Cell Signaling Technologies	2535	1:1000
Anti-eIF2α	Santa Cruz	sc-11386	1:2000
Anti-p(S51)-eIF2α	Cell Signaling Technologies	3597	1:2000
Anti-GCN2	Cell Signaling Technologies	3302	1:1000
Anti-p(T899)-GCN2	Abcam	ab75836	1:1000
Anti-mTOR	Cell Signaling Technologies	2972	1:1000
Anti-p(S2448)-mTOR	Cell Signaling Technologies	2971	1:1000
Anti-rpS6	Cell Signaling Technologies	2217	1:1000
Anti-p(S235/236)-rpS6	Cell Signaling Technologies	4858	1:1000
Anti-ULK1	Cell Signaling Technologies	8054	1:2000
Anti-p(S757)-ULK1	Cell Signaling Technologies	14202	1:2000
Anti-rabbit	Jackson ImmunoResearch	111-035-003	1:20000
