# Supplementary material for: Activation and execution of the hepatic integrated stress response by dietary essential amino acid deprivation is amino acid specific
Source: FASEB J. 2022 Jun 12;36(7):e22396. doi: 10.1096/fj.202200204RR (PMC9204950; doi:10.1096/fj.202200204RR)
Supplement: Supplementary file 8 — Table S4 [file FSB2-36-0-s008.pdf]

Table S4. Male mice provided diets devoid of leucine or the sulfur amino acids for six hours showed altered serum amino acid concentrations.

Amino acid	WT.Ctrl		WT.LD		WT.SAAD		Gcn2KO.Ctrl		Gcn2KO.LD		Gcn2KO.SAAD		Statistical effect
	Mean (pmol/μL)	SD (pmol/μL)	Mean (pmol/μL)	SD (pmol/μL)	Mean (pmol/μL)	SD (pmol/μL)	Mean (pmol/μL)	SD (pmol/μL)	Mean (pmol/μL)	SD (pmol/μL)	Mean (pmol/μL)	SD (pmol/μL)	
Alanine	1062.42	59.26	1118.18	118.07	1104.17	584.20	774.89	47.06	1030.02	288.81	936.94	183.81	None
Arginine	184.24	16.63	147.52	10.99	170.71	24.43	148.89	24.16	148.93	23.44	138.87	11.79	p<0.05 (genotype)
Asparagine	81.87	14.69	65.61	10.04	76.25	42.57	59.82	14.81	76.21	20.94	65.64	14.89	None
Aspartic acid	13.73	2.79	10.23	4.40	17.22	10.32	11.10	1.44	12.96	2.05	15.07	7.91	None
Cystine	29.57	6.12	28.45	9.46	15.75	7.24	23.98	4.30	23.28	2.85	16.73	8.02	p<0.05 (diet)
Glutamic acid	57.09	27.71	49.06	8.80	58.90	33.42	68.82	24.37	56.88	21.94	174.28	109.27	p<0.05 (genotype)
Glutamine	994.99	193.72	1060.91	74.75	1084.95	188.85	900.54	89.67	1124.16	209.48	1118.56	497.07	None
Glycine	1721.32	191.29	1588.19	148.83	1548.77	296.70	1369.12	129.11	1564.45	358.44	1892.93	353.42	None
Histidine	90.71	17.66	72.01	30.92	101.23	63.24	60.06	5.85	71.07	11.69	58.65	19.88	None
Hydroxy proline	490.79	440.45	316.76	29.77	311.76	104.65	267.77	35.42	289.25	30.19	499.15	509.10	None
Isoleucine	148.74	22.98	275.95	76.02	147.30	46.01	130.31	34.98	290.79	46.35	131.90	20.87	p<0.001 (diet)
Leucine	211.30	20.31	45.50	10.56	207.96	50.07	188.68	48.11	43.34	6.11	201.17	34.50	p<0.001 (diet)
Lysine	488.96	144.73	396.56	130.78	404.78	179.66	276.75	83.78	240.58	63.03	225.57	74.05	p<0.01 (genotype)
Methionine	259.72	15.69	242.78	14.51	43.55	13.20	248.34	73.93	240.19	61.85	46.67	17.03	p<0.001 (diet)
Phenylalanine	111.49	11.23	98.95	15.34	133.80	58.86	89.74	20.22	93.60	6.43	99.86	13.04	None
Proline	423.72	71.16	332.13	89.63	285.55	114.04	472.44	61.84	414.80	31.92	392.79	140.86	None
Serine	236.66	12.33	207.34	17.71	196.86	53.94	197.51	21.07	244.25	85.57	249.07	86.51	None
Threonine	480.27	38.55	363.78	129.89	340.89	46.28	325.81	77.70	341.53	65.08	318.27	74.34	None
Tryptophan	103.51	19.71	97.68	33.11	126.24	36.04	87.89	25.36	90.15	9.50	111.63	38.93	None
Tyrosine	129.89	7.99	216.78	58.00	183.05	20.47	125.42	37.07	147.21	14.16	157.76	43.71	p<0.05 (diet)
Valine	328.27	30.12	630.22	133.66	374.37	130.69	325.35	79.46	628.76	60.84	338.64	47.58	p<0.001 (diet)

Serum amino acid concentrations in male wild-type (WT) and *Gcn2* knockout (*Gcn2* KO) mice refed with either a control (Ctrl), leucine devoid (LD) or sulfur amino acid devoid (SAAD) diet for six hours. n = 3-4/group. Displayed p-values were determined by two-factor ANOVA. “None” signifies no statistical difference at α = 0.05. Values are displayed as mean and standard deviation (SD).
