# Supplementary material for: Activation and execution of the hepatic integrated stress response by dietary essential amino acid deprivation is amino acid specific
Source: FASEB J. 2022 Jun 12;36(7):e22396. doi: 10.1096/fj.202200204RR (PMC9204950; doi:10.1096/fj.202200204RR)
Supplement: Supplementary file 9 — Table S5 [file FSB2-36-0-s002.pdf]

Table S5. Male mice provided diets devoid of leucine or the sulfur amino acids for six hours showed altered liver intracellular amino acid concentrations.

Amino acid	WT.Ctrl		WT.LD		WT.SAAD		Gcn2KO.Ctrl		Gcn2KO.LD		Gcn2KO.SAAD		Statistical effect
	Mean (pmol/mg)	SD (pmol/mg)	Mean (pmol/mg)	SD (pmol/mg)	Mean (pmol/mg)	SD (pmol/mg)	Mean (pmol/mg)	SD (pmol/mg)	Mean (pmol/mg)	SD (pmol/mg)	Mean (pmol/mg)	SD (pmol/mg)	
Alanine	9298.34	2803.83	12030.79	3096.57	7872.85	2571.67	9680.94	2577.32	6887.99	739.51	9199.36	4183.21	None
Arginine	17.72	8.82	18.97	12.84	27.81	18.85	21.09	7.00	24.62	8.58	28.78	8.10	None
Asparagine	140.90	30.01	110.14	51.32	148.17	40.96	98.85	8.23	101.30	33.70	248.00	175.51	None
Aspartic acid	249.76	85.57	231.48	77.36	248.16	82.90	224.52	72.11	210.54	58.74	238.29	231.94	None
Cystine	BDL	BDL	BDL	BDL	BDL	BDL	BDL	BDL	BDL	BDL	BDL	BDL	N/A
Glutamic acid	1451.66	599.35	2055.71	547.89	2030.16	997.37	2639.28	390.40	2030.53	208.22	3068.87	1438.01	None
Glutamine	6805.15	2067.42	5145.87	2853.43	6572.38	2561.01	5705.46	1684.76	6014.89	2468.04	10966.54	5330.39	None
Glycine	17929.22	5726.95	16528.42	3436.31	18520.57	5760.00	17927.89	2761.17	13565.10	2529.69	23733.61	11182.59	None
Histidine	531.21	148.29	557.67	118.16	662.41	269.65	510.69	61.35	463.09	90.13	332.19	92.56	p<0.05 (genotype)
Hydroxy proline	88.28	15.57	85.43	23.03	338.67	390.73	79.04	12.24	70.53	18.39	182.66	119.75	p<0.01 (diet)
Isoleucine	209.55	55.63	388.94	74.21	276.16	47.83	228.10	8.26	325.30	7.63	252.75	82.17	p<0.001 (diet)
Leucine	353.03	100.54	147.18	58.35	437.16	73.40	365.83	11.60	101.68	36.63	393.62	128.78	p<0.001 (diet)
Lysine	403.17	138.69	741.67	319.88	616.24	268.29	338.55	192.88	390.07	119.82	408.78	107.85	p<0.05 (genotype)
Methionine	55.26 (ab)	17.23	98.60 (a)	26.75	41.34 (b)	16.36	89.84 (a)	22.16	61.69 (ab)	15.41	68.99 (ab)	6.53	p<0.01 (interaction)
Phenylalanine	108.70	36.71	116.73	37.88	119.94	41.57	94.06	9.94	83.70	7.70	98.73	27.44	None
Proline	67.88	10.59	67.23	11.02	62.36	8.87	74.47	2.72	74.98	2.81	154.83	162.82	None
Serine	760.38	181.37	862.27	191.53	799.20	251.15	721.01	97.23	582.27	41.67	974.43	393.69	None
Threonine	894.16	240.49	1095.52	317.15	518.83	103.24	985.19	109.06	923.19	184.97	514.99	182.14	p<0.001 (diet)
Tryptophan	28.99	5.41	27.03	7.32	44.25	10.17	24.79	1.55	26.60	4.50	44.35	23.13	p<0.01 (diet)
Tyrosine	117.31	25.38	205.20	50.88	160.36	55.64	132.87	45.02	116.26	18.21	133.68	56.85	None
Valine	406.51	113.62	862.53	96.30	722.77	334.24	480.73	41.06	693.68	40.61	595.36	185.41	p<0.01 (diet)

Liver amino acid concentrations in male wild-type (WT) and *Gcn2* knockout (*Gcn2* KO) mice refed with either a control (Ctrl), leucine devoid (LD) or sulfur amino acid devoid (SAAD) diet for six hours. n = 3-4/group. Displayed p-values were determined by two-factor ANOVA. Means without shared letters were statistically different at α = 0.05, as determined by pair-wise t-tests with Bonferroni correction for multiple comparisons. “None” signifies no statistical difference at α = 0.05. Values are displayed as mean and standard deviation (SD).
